# Editorial: Writing a manuscript for the JACMP

**DOI:** 10.1120/jacmp.v10i2.3083

**Published:** 2009-04-28

One of the most difficult tasks facing the clinical medical physicist who has completed work on a clinical problem and wishes to report on that activity in the JACMP (or any other publication, for that matter) is writing the manuscript. Medical physicists tend to be good, even very good, at a host of other job‐related tasks, such as evaluating technologies and transferring them into the clinic, designing and implementing quality assurance programs, assessing the safety and efficacy of clinical procedures, as well as many other tasks. However, we sometimes lack the ability to communicate the results of our work to others in a clear, concise, and understandable manner that would be suitable for publication in our journal. As a consequence, information that may be of use to the practicing clinical medical physicist may not necessarily be published and may not necessarily be shared with the medical physics community. In the few pages of this Editorial, I hope to share with you some thoughts that might help you jump‐start the process of describing in words some of your clinical advancements. It is my hope that you might be motivated to share your knowledge with your colleagues and submit a manuscript to the JACMP.

I want to acknowledge the source of some of the material that is being presented here as coming from the Department of Scientific Publications, The University of Texas M. D. Anderson Cancer Center, my home institution. Staff in this Department have been an invaluable resource in providing editorial review for scientific publications of our faculty and staff.

First, some general words of advice in preparing a manuscript for publication:

- Know your audience. The JACMP is directed toward the medical physicist practicing in the clinic. Most of our audience hold advanced degrees in physics, engineering, or medical physics, and have some degree of clinical knowledge and experience. Target your manuscript to this audience. Assume that your reader knows something about medical physics, but do not assume the reader possesses the expertise in the narrow field that you are addressing with your manuscript. Part of your task as an author is to teach your colleagues.
- Know the purpose of the journal. The JACMP is a clinical journal, so make the clinical applications clear. If the topic of your manuscript is not clinical, but research, the manuscript should be directed toward a scientific journal such as *Medical Physics* or *Physics in Medicine and Biology*. If the topic of your manuscript addresses issues that are more medical, perhaps a journal such as the *International Journal of Radiation Oncology Biology Physics* or *Radiology* might be a better fit.
- Make sure you are using good English. This may be a difficult issue for many authors of JACMP manuscripts who are from non–English‐speaking countries. If English is not your native language, try to get some editorial review from a native English speaker before submitting your manuscript. Editorial review is a good idea even if English is your native language, as a good editor can often help you in making your points clear.
- Write simply. Short declarative sentences are most effective in getting your point across to the reader. Your goal is to convey information, not to impress your audience with your erudition.

Fortunately, writing for a scientific or clinical journal is relatively formulaic. The creativity lies in the science and not in the writing (although around 1970, a manuscript written in rhymed couplets was published in a chemistry journal). It is relatively straightforward to set up a writing template and fll in the blanks, creating a clear, well‐written manuscript.

To begin with, divide your manuscript into the following sections:

- Abstract
- Introduction
- Methods and Materials
- Results
- Discussion
- Conclusions
- References, figure captions, table listings

The Abstract is a summary of your manuscript, and is probably best written after the rest of the manuscript has been completed. Follow the “8‐sentence” rule in writing your Abstract. Devote two sentences to the purpose of your study, two sentences to the methods and materials, two sentences to your results, and two sentences to your conclusions. In these eight sentences you have summarized your story. Details can be found in the body of your text, not in the Abstract. Limit the information in the Abstract only to information that is in your manuscript; do not include information in the Abstract that is not in the manuscript.

The Introduction prepares the reader for the details of the work. In the Introduction, you provide the motivation for your study. Include enough background to support the reason why you did the study; the Introduction is not meant to be a review article or even be a comprehensive summary of the literature. Specifically identify the problem you were trying to solve or the gap in knowledge your study was intended to fill. Follow the identification of the problem with a short (one or two sentence) summary of your methodology, and conclude your Introduction with a brief description of the answer to your question.

The Methods and Materials section of your manuscript is perhaps the easiest part of the manuscript to write. Simply provide a narrative of what you have done with enough detail to permit the reader to duplicate your study. Write in the past tense using an active voice, with phrases such as “We determined&,” “We measured &,” “We used &” In this section, you describe to the reader what you did and explain to the reader why you did what you did.

In the Results section, you present your results. This is the section in which most of the figures and tables go, but use the text to highlight what you want the reader to know. A former colleague of mine would generate figures and tables of all his results, post them on his wall, and then decide what should go into his manuscripts. Do not repeat the description of your methodology in this section; even more importantly, state only your results, and do not elaborate or interpret the results.

The Discussion is the section in which you interpret your results. Do not repeat your background information, methods, or results. Rather, show how your work relates to existing literature. In this section, you can also critique your study. Identify the strengths and the limitations of your study. Show the reader how the information you determined in your study can be generalized. Identify some of the implications of your work. Finally, you might want to give some indication of where you plan to go next with your work.

The last section of your manuscript is the Conclusions section. Keep this section brief; it should be a 1‐ or 2‐paragraph summary of your conclusions. Base your conclusions on your work and other work that has been done, but do not speculate, as speculation belongs in the Discussion section.

Finally, feel free to take advantage of the fact that the JACMP is an electronic journal and make use of the Supplementary Files. In these files you can place data sets, video clips, or anything else that you would not be able to insert into the body of a journal article.

I hope these comments may prompt you into sharing some of your clinical developments with the JACMP readership. Good luck in writing your next submission to the JACMP, and I will be looking forward to seeing your submission.

George Starkschall, PhD

Editor‐in‐Chief

May 15, 2009

